# Current status and future development of solvent-based carbon capture

**DOI:** 10.1007/s40789-017-0159-0

**Published:** 2017-02-27

**Authors:** Eni Oko, Meihong Wang, Atuman S. Joel

**Affiliations:** 1 Department of Chemical and Biological Engineering, University of Sheffield, Sheffield, S1 3JD UK; 2 School of Engineering, University of Hull, Hull, HU6 7RX UK

**Keywords:** Post-combustion CO_2_ capture (PCC), Chemical absorption, Solvents, Process intensification

## Abstract

Solvent-based carbon capture is the most commercially-ready technology for economically and sustainably reaching carbon emission reduction targets in the power sector. Globally, the technology has been deployed to deal with flue gases from large scale power plants and different carbon-intensive industries. The success of the technology is due to significant R&D activities on the process development and decades of industrial experience on acid gas removal processes from gaseous mixtures. In this paper, current status of PCC based on chemical absorption—commercial deployment and demonstration projects, analysis of different solvents and process configurations—is reviewed. Although some successes have been recorded in developing this technology, its commercialization has been generally slow as evidenced in the cancellation of high profile projects across the world. This is partly due to the huge cost burden of the technology and unpredictable government policies. Different research directions, namely new process development involving process intensification, new solvent development and a combination of both, are discussed in this paper as possible pathways for reducing the huge cost of the technology.

## Introduction

Carbon capture and storage (CCS) is considered the most sustainable and economic option for cutting down CO_2_ emissions from large stationary sources such as coal-fired power plants and other carbon-intensive industries (e.g. refineries, steelworks, cement plants) due to the trilemma of ensuring clean, secure and affordable energy sources (IPCC 2014). CCS technology involves capturing CO_2_ from these sources and transporting them to underground storage sites, namely saline aquifer and depleted oil and gas reserves, where they are either stored permanently and prevented from entering the atmosphere or used for enhanced oil recovery (EOR) purposes (IPCC 2014). Without CCS, cost of CO_2_ emission reduction in these sectors may be up to 70% more (CCSA 2011).

**Fig. 1:**
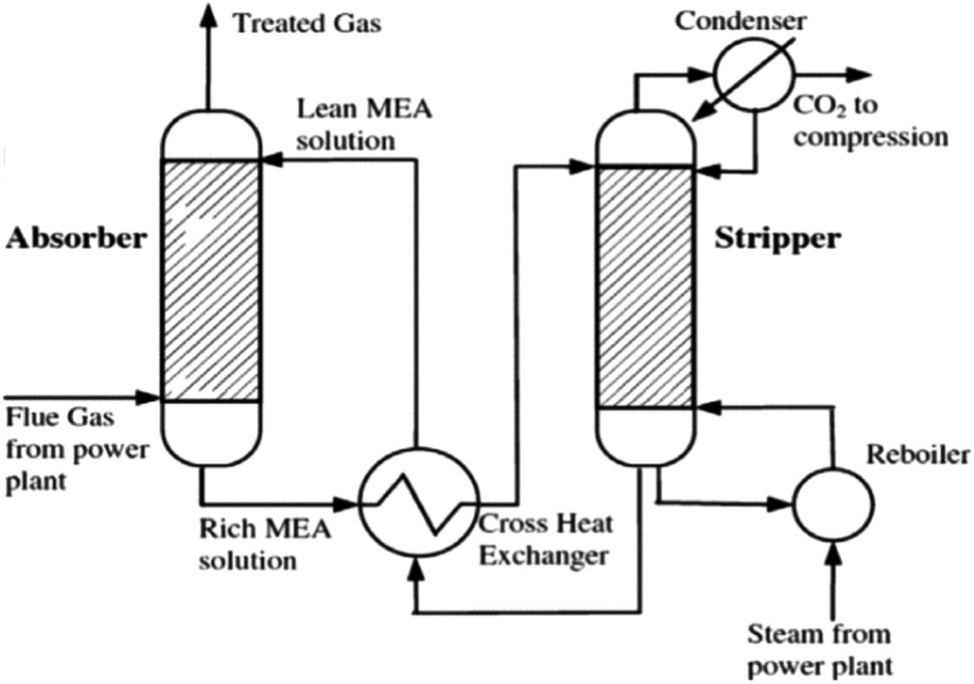
Diagram of PCC process based on chemical absorption (IPCC 2005)

CCS can be implemented using different approaches, namely post-combustion (PCC), pre-combustion and oxy-fuel capture (Wang et al. 2011). In the different approaches, there are different processes for separating CO_2_ from gas mixtures such as chemical absorption, physical absorption, adsorption and membrane separation. Other emerging processes such as chemical looping (Olaleye and Wang 2014) and calcium looping (Blamey et al. 2010) also have good potentials. Implementing CCS through PCC based on chemical absorption (Fig. 1) offers some benefits compared to other processes (IEAGHG 2014). These include reliance on established technologies and capacity to be retrofitted to existing power plants/industrial plants with minimal modifications. PCC processes based on chemical absorption (with conventional amine solvents) is also currently at a technology readiness level (TRL) of 6–8 (TRL 6—fully integrated pilot tested in a relevant environment, TRL 7—subscale demonstration, fully functional prototype, TRL8—commercial demonstration) (IEAGHG 2014). Consequently, many first generation CCS projects are expected to be implemented through PCC based on chemical absorption (Wang et al. 2011). A detailed description of the process is given in Wang et al. (2011).

Although several CCS projects using PCC based on chemical absorption have been completed in the past, the capital and operating cost of the process remains unacceptably high and substantial research efforts have been devoted to address this need. The aim of this paper is to provide an update on current and predicted future research and development (R&D) activities on PCC based on chemical absorption. These include pilot plant testing, demonstration project overview, and other commercial activities, assessment of the process configurations (flowsheet development) and different solvents used in the process. These discussions set this paper apart from other related reviews such as Wang et al. (2011) and Boot-Handford et al. (2014).

## Process configurations

**Fig. 2:**
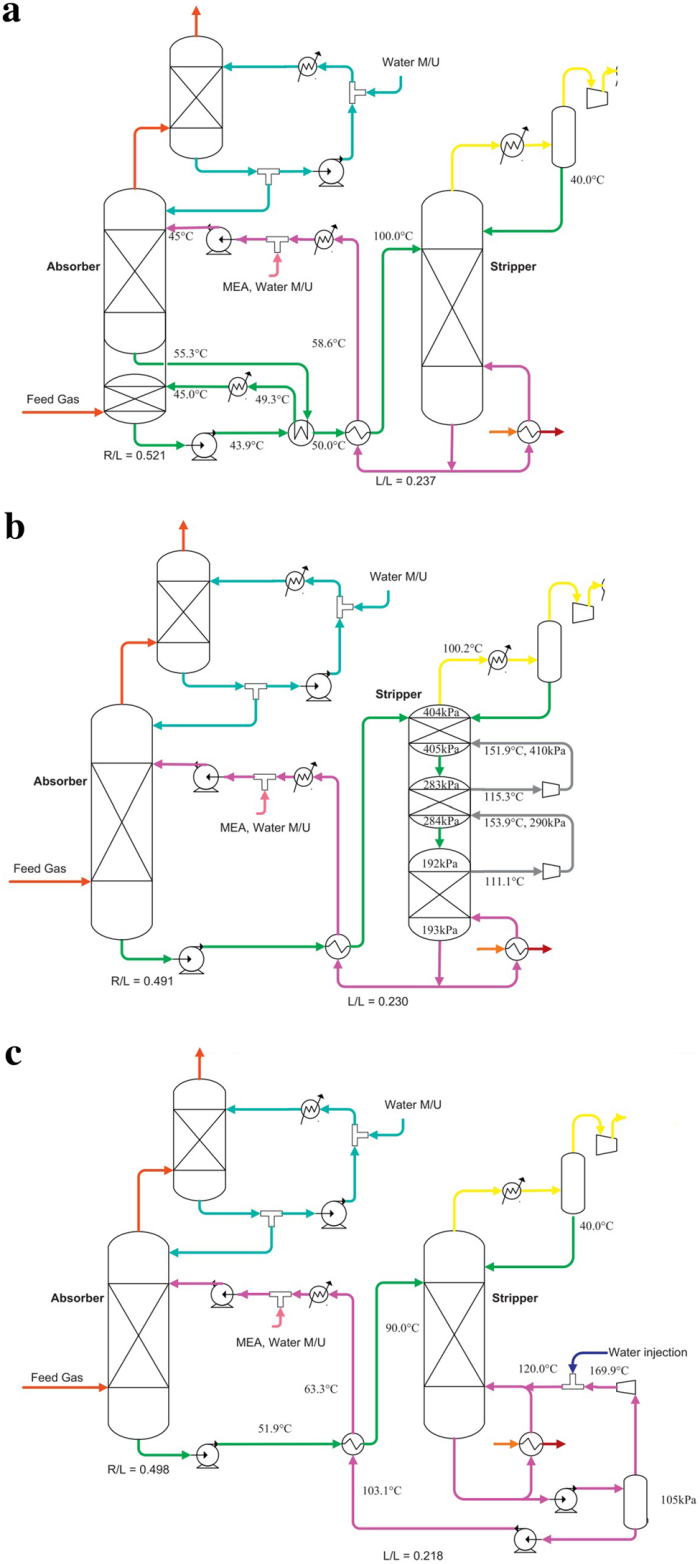
Alternative configurations **a** absorber inter-cooling, **b** multi-pressure stripping, **c** split solvent flow, **d** lean solvent flash (Ahn et al. 2013)

Alternative process configurations have been developed by adding extra equipments (e.g. heat exchangers, compressors, flash drum etc.) to the conventional process (Fig. 1). Typical examples include configurations involving absorber inter-cooling, multi-pressure stripping and split-flow (Fig. 2) among others (Fisher et al. 2007; Ahn et al. 2013; Boot-Handford et al. 2014). Thermodynamic analysis of these configurations with 30 wt% monoethanolamine (MEA) solvent shows that they are more energy efficient than the conventional configuration (see Table 1). However, due to the extra instrumentation/equipment, their capital costs will be predictably higher. The Boundary Dam commercial PCC plant in Canada incorporates an absorber inter-cooler configuration (IEAGHG 2015).

**Table 1:** Energy consumptions for different process configurations (Ahn et al. 2013)

Configuration	Reboiler duty (MJ_th_/kg_CO2_)	Total energy demand (MJ_e_/kg_CO2_)
Conventional	3.52	1.380
Absorber intercooling	3.11	1.257
Multi-pressure stripping	3.17	1.353
Split amine flow	3.09	1.252
Lean amine flash	2.76	1.220

## Solvents

MEA solution (30 wt% or less MEA) is generally considered a benchmark solvent for PCC based on chemical absorption process. The oldest commercial PCC processes, Kerr-McGee/ABB Lummus Crest process and Fluor Daniel’s Econamine FG process, use 20 wt% and 30 wt% MEA solutions as solvents respectively (Rao et al. 2004). MEA has rapid kinetics but requires high regeneration energy (in the range of 3.2–4.2 GJ/tonne CO_2_); the host power plant could be de-rated by more than one-third of its capacity when integrated to a PCC plant with MEA solution as solvent (Fisher et al. 2007). CO_2_ loaded MEA solution is also very corrosive and degrades rapidly. MEA solution also entails high solvent circulation rate which leads to large equipment sizes and high energy consumption.

**Fig. 3:**
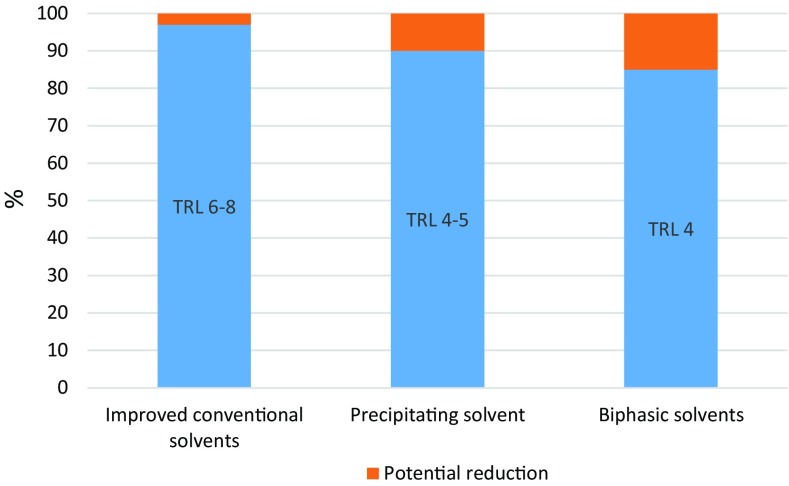
LCOE scenario for different solvents compared to MEA solvent (IEAGHG 2014)

These drawbacks of MEA has been addressed through development of new solvents which include mixed amine solvents such as mixtures of MEA and MDEA, AMP and PZ among others (Dubois and Thomas 2012), ammonia-based solvents (Darde et al. 2010), amino acid solvent (Brouwer et al. 2005), biphasic solvents (Raynal et al. 2011) and ionic liquid-based solvents (Boot-Handford et al. 2014; Zacchello et al. 2016). The new solvents have shown great potential. For instance, biphasic solvents require about 50% less regeneration energy and have about four times cyclic loading capacity compared to MEA (Zhang et al. 2013). Existing commercial PCC processes (see Table 3) use solvents formulated with these new solvents. Notwithstanding the successes with solvent development, analysis of PCC process using improved solvents such as improved conventional solvents (i.e. mixed amine), precipitating solvents (i.e. amino acid) and biphasic solvents among others shows that levelized cost of electricity (LCOE) for a power plant integrated with the PCC process will only reduce by less than 20% compared to a scenario with MEA solvent (see Fig. 3). The solvents that have shown the greatest potentials namely biphasic solvents are still at development phase with TRL of 4.

## Research and development (R&D) activities worldwide

### Pilot/demonstration PCC plants

Globally, R&D activities include laboratory and field scale pilot plants as summarised in Wang et al. (2011) and successful trials of demonstration PCC plants integrated to live power plants (Table 2). The demonstration plants integrated to the power plants via flue gas slipstream have resulted in the development of commercial PCC processes (Table 3).

**Table 2:** Summary of trials of PCC based on chemical absorption process integrated to live power plants

Project	Location	Consortium	Cost	Capacity	Year
Pleasant Prairie (Alstom 2009)	Wisconsin, USA	Alstom Power/Electric Power Research Institute/We Energies	US$8.6 M	15,000 tCO_2_/year	2008–2009
E.ON Karlshamn (MIT 2016a)	Malmo, Sweden	E.ON Thermal Power/Alstom Power	US$15 M	15,000 tCO_2_/year	2009–2010
AEP Mountaineer (Alstom 2011)	West Virginia, USA	American Electric Power (AEP)/Alstom Power/RWE/NETL/Battelle Memorial Institute	US$668 M	100,000 tCO_2_/year	2009–2011
Brindisi (Mangiaracina 2011)	Brindisi, Italy	Enel and Eni.	€20 M	8000 tCO_2_/year	2010–2012
Plant Barry (MIT 2016b)	Alabama, USA	Southern Energy/Mitsubishi Heavy Industries/Southern Company/U.S. DOE’s Southeast Regional Carbon Sequestration Partnership and EPRI	Unknown	500 tCO_2_/day	2011–2012
Gaobeidian (Ju 2015)	Beijing, China	Huaneng Power Group/CSIRO	Unknown	3000 tCO_2_/year	2008 to present
Shidongkou (Ju 2015; MIT 2016d)	Shanghai China	Huaneng Power Group	US$24 M	120,000 tCO_2_/year	2010 to present
Shenhua (Ju 2015)	Inner Mongolia, China	Shenhua Group	Unknown	100,000 tCO_2_/year	2010–2014
Sinopec (Ju 2015)	Shangdong, China	Sinopec Group	Unknown	40,000 tCO_2_/year	2010–2012
Boryeong (Lee et al. 2015; MIT 2016e)	Boryeong, S. Korea	Korea Electric Power Company (KEPCO)	US$42 M	2 tCO_2_/day (Phase 1) 200 tCO_2_/day (Phase 2)	2010–2013
Wilhelmshaven (Radgen et al. 2014)	Bremen, Germany	Fluor/E.ON Kraftwerke	Unknown	70 tCO_2_/day	2012–2014
CCSPilot100+ (Fitzgerald et al. 2014)	Ferrybridge, UK	SSE/Doosan Babcock/Vattenfall	£21 M	100 tCO_2_/day	2012–2013
ECO_2_ (Powerspan 2016)	Burger	First Energy/Powerspan/Ohio Coal Development Office	Unknown	20 tCO_2_/day	2008–2010
Aberthaw (MIT 2016f)	Wales	RWE npower/CanSolv Technologies Inc.	Unknown	50 tCO_2_/day	2013–2014
Pikes Peak (MIT 2016g)	Saskatchewan	Husky Energy Inc./CO_2_ Solutions	US$12.13 M	15 tCO_2_/day	2015
EDF (Chopin 2014)	Le Havre, France	EDF/Veolia/Alstom Power/Dow Chemical	€22 M	25 tCO_2_/day	2013–2014

**Table 3:** Commercial PCC based on chemical absorption processes

PCC process	Developer	Solvent	Demonstration	Commercial project
CanSolv^®^ (Shaw 2009)	Shell	Amine-based	TCM Norway Aberthaw PCC Wales	Boundary Dam Canada (Operational) Bow City Canada (Planning)
Advanced Capture Process (Nustad 2012)	Aker Clean Carbon	Amine-based	TCM Norway	Longannet UK (Cancelled) Porto Tolle Italy (Cancelled)
PostCap™ (Siemens 2015)	Siemens	Amino acid salt	TCM Norway Big Bend PCC Florida	ROAD Netherlands (Planning) Masdar Abu Dhabi (Planning)
Econamine FG Plus^SM^ (Reddy et al. 2008)	FLOUR	Amine-based	TCM Norway Wilhelmshaven PCC Germany	Trailblazer, Texas (Cancelled)
Advanced Amine Process (Chopin 2014)	Alstom Power/Dow Chemical	DOW UCARSOL™ FGC 3000	EDF PCC Le Havre, France Charleston PCC, West Virginia	Elektownia Belchatow, Poland (Planning) GETICA Romania (on-hold)
CAP^®^ (Alstom 2009, 2011; MIT 2016a)	Alstom Power	Chilled ammonia	TCM Norway Pleasant Prairie PCC Milwaukee Karlshamn PCC Sweden Mountaineer CCS Phase I, West Virginia	AEP Mountaineer CCS Phase II, West Virginia (Cancelled) Project Pioneer Alberta (Cancelled)
KM-CDR™ (MIT 2016b)	MHI/KEPCO	KS-1 (Hindered amine)	Plant Barry, Alabama Plant Yates, Georgia	Petro-Nova CCS, Texas (On-going)
ECO_2_™ (Powerspan 2016)	Powerspan	Amine-based	Burger PCC, Ohio	
HTC (HTC Purenergy 2016)	HTC Purenergy/Doosan Babcock	Amine-based	International Test Centre, Canada	Antelope Valley CCS, North Dakota
CO_2_ Solution (MIT 2016c)	CO_2_ Solutions Ltd	Enzyme-based solvent	Pikes Peak South PCC, Saskatchewan, Canada	
DMX™ (Raynal et al. 2013)	IFPEN/PROSERNA	Biphasic solvent	ENEL’s Brindisi Pilot PCC, Italy	
RSAT™ (Gayheart et al. 2012)	Babcock and Wilcox	OptiCap^®^		

Another major milestone in PCC development is the European CO_2_ Test Centre Mongstad (TCM) in Norway. TCM is a specialist centre for testing different PCC technologies, namely Chilled Ammonia and Amine-based PCC processes (TCM 2016). TCM was developed by a consortium involving Gassnova, Statoil, Sasol and Shell, and is estimated to have cost about US$1.02 billion. Several PCC technologies based on chemical absorption have been tested successfully at TCM, namely Alstom’s Chilled Ammonia Process (CAP™), Aker’s Clean Carbon, Shell’s CanSolv™ and Siemens’ PostCap™ among others.

A generic independent verification protocol (IVP) developed by Electric Power Research Institute (EPRI) has been used as an independent benchmark for assessing the process performance in some of the pilot plant tests (Alstom 2009, 2011; Chopin 2014; Thimsen et al. 2014; MIT 2016b). In most of the tests, CO_2_ capture level up to 90% and average steam consumption of 1 ton/ton CO_2_ were reportedly achieved. During the tests, CO_2_ captured is either vented into the atmosphere (Alstom 2009, 2011; Chopin 2014), transported and stored underground (Ju 2015; MIT 2016b) or sold to beverage industries (Ju 2015). Investigations carried out during the pilot plant studies include (1) profiling different solvents; (2) scale-up procedure; (3) solvent degradability; (4) corrosion studies; (5) operation study and (6) process energy efficiency (Chi and Rochelle 2002; Uyanga and Idem 2007; Davis and Rochelle 2009; Kittel et al. 2009; Mangalapally et al. 2009; Alstom 2011; Faber et al. 2011; Seibert et al. 2011; Rabensteiner et al. 2014).

### Modelling and simulation

#### Model development

Dynamic models of stand-alone absorber (Posch and Haider 2013; Kvamsdal et al. 2009; Kvamsdal and Rochelle 2008; Khan et al. 2011; Lawal et al. 2009a) and stand-alone stripper (Lawal et al. 2009b; Zaii et al. 2009) which are the main components of the PCC process based on chemical absorption are available in literature. The dynamic models of the complete process including the absorber and stripper are also available (Lawal et al. 2010; Harun et al. 2011; Gáspár and Cormoş 2011; MacDowell et al. 2013; Flø et al. 2015). Two-film theory is used in most of the models to represent rate-based mass transfer. Some papers such as Posch and Haider (2013) used equilibrium-based approach which involves approximate mass transfer calculations. Comparative assessment of rate-based and equilibrium-based models of the process in Lawal et al. (2009a, b) showed that rate-based models give better predictions.

Reaction kinetics are neglected in some of the models (Lawal et al. 2009a, b; Zaii et al. 2009; Lawal et al. 2010; Biliyok et al. 2012; MacDowell et al. 2013). This is based on the assumption that the reactions have rapid kinetics and are able to attain equilibrium. The assumption is valid for cases involving solvents with rapid kinetics such as MEA (Kenig et al. 2001). Reaction kinetics have also been described more accurately by introducing an enhancement factor (Kvamsdal and Rochelle 2008; Kvamsdal et al. 2009; Gáspár and Cormoş 2011; Harun et al. 2011; Khan et al. 2011; Flø 2015) or using actual reaction kinetics model (Aboudheir et al. 2003; Posch and Haider, 2013).

Dynamic models by Lawal et al. (2009a, b), Lawal et al. (2010), Gáspár and Cormoş (2011) and MacDowell et al. (2013) were validated at steady state conditions only over limited conditions at pilot scale due to lack of experimental data for detailed validations as at the time of their publications (Chikukwa et al. 2012). Validation of dynamic models under state steady state and dynamic conditions have also been attempted by Biliyok et al. (2012). The validation results showed good agreement.

The models were used to study the sensitivities of key process variables (e.g. capture level, solvent loading) at different operating conditions under steady state and dynamic scenario and phenomena such as temperature bulge in the absorber (Kvamsdal and Rochelle 2008). More extensive pilot PCC plant data logs (steady state and dynamic) acquired from different PCC demonstration plants namely Brindisi CO_2_ capture pilot plant (Italy) and TCM (Norway) are now available (Flø 2015). Brindisi and TCM are significantly large scale compared to the pilot PCC plant at University of Texas, Austin (Dugas 2006), the capture facility at NTNU laboratory in Gløshaugen, Trondheim and the capture facility at SINTEF laboratory in Tiller, Trondheim among others where data used for validating most models were obtained (Lawal et al. 2010; Biliyok et al. 2012; Flø et al. 2015). The new data have been used to validate dynamic models by Flø et al. (2015) under steady state and dynamic conditions.

#### Commercial tools for model development

Available commercial tools for developing PCC models include Aspen Plus/Custom Modeller developed by Aspen Technology Inc., USA (Zhang et al. 2009) and gCCS developed by a consortium headed by PSE Ltd, UK (Rodríguez et al. 2014). Both applications are CAPE-OPEN compliant and supports rigorous thermodynamic models, namely eNRTL and SAFT-VR among others. The gCCS platform also supports modelling and simulation of all components of the CCS chain (i.e. power plant, solvent-based capture plant, CO_2_ compression and pipeline transport, and underground storage). It was selected to be used for the Front-End Engineering (FEED) study of the planned commercial-scale Peterhead CCS in Scotland (PSE 2014) which is now suspended indefinitely (BBC 2015).

## Commercial deployment

Commercial CO_2_ absorption/stripping plants within CCS context are widely deployed in industries; Sleipner CCS Norway, In Salah CCS Algeria, Snøhvit CCS Norway (Eiken et al. 2010; Ringrose et al. 2013) and more recently, Gorgon CCS Australia and Quest CCS Canada (Shell 2015). However, in power plants, Boundary Dam CCS Canada is the only operational CCS project that is based on chemical absorption process (Stéphenne 2014; SaskPower 2016). The plant was built at a total cost of US$1.3 Billion to capture about one million tonnes of CO_2_ per annum using Shell’s CanSolv^®^ PCC process from a re-built 139 MWe (gross) coal-fired power plant. Based on expected revenue from sales of CO_2_, sulphuric acid and fly ash sales, SaskPower claims that the LCOE of the host power plant is comparable to that of a Natural Gas Combined Cycle (NGCC) Power Plant (Daverne 2012; Clark and Herzog 2014).

There have been mixed reports about the success of the Boundary Dam project. SaskPower claims that the plant achieves up to 90% capture level when operational (SaskPower 2015). Media reports suggest otherwise claiming that the plant only achieves about 45% capture level (ENDCOAL 2015; Reneweconomy 2015). Regular mechanical failures have also been reported (Power 2015) and this limited the plant availability to about 40% during some period (Reneweconomy 2015). During this period, SaskPower could not deliver on their CO_2_ supply agreement with Cenovus Energy. Recently, SaskPower confirmed nearly 100% availability for the months of Dec. 2015 and Jan. 2016. It projected an average 85% availability for the next year (Estevan 2016).

Other power plant-based carbon capture projects across the world such as Petra-Nova CCS, Texas (to be launched later in 2017) are beset with challenges ranging from unfavourable government policies, lack of economic incentives and huge capital cost.

## Future research directions

### New process

**Fig. 4:**
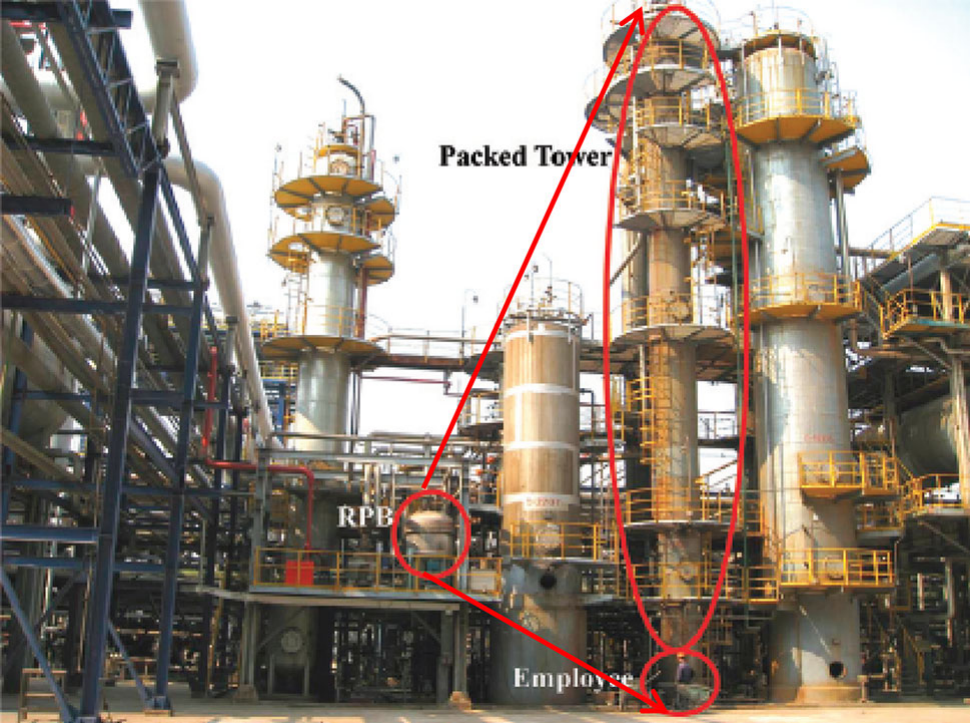
Commercial demonstration of RPB for desulfurization (Qian et al. 2012)

Currently, the process comprises of large absorber and stripper packed beds which contribute significantly to plant footprint, capital and operating cost. Through process intensification (PI), the size of the absorber and strippers can be reduced significantly (Reay et al. 2013; Wang et al. 2015). In PI equipments such as rotating packed beds (RPBs), the liquid and gas flows are subjected to intense centrifugal acceleration which is many times the gravitational acceleration in conventional packed beds. This allows higher flooding rate and lower interfacial mass transfer resistance resulting in significant reduction in the packed bed sizes. Recent studies have demonstrated prospects of replacing conventional packed beds in PCC with RPB (Agarwal et al. 2010; Joel et al. 2014; Thiels et al. 2016). Agarwal et al. (2010) and Joel et al. (2014) reported 7 and 12 times packing volume reduction respectively for separate cases involving replacement of conventional packed bed with RPB for Absorbers in PCC based on chemical absorption. RPBs have been demonstrated successfully in industry for natural gas desulphurization applications (Fig. 4) (Qian et al. 2012). However, application of RPBs in PCC is still at an early stage of development with many issues, namely scale-up, flooding limit, operating performance and pressure drop, that are yet to be properly understood. This is the focus of an engineering and physical sciences research council (EPSRC) funded research consortium in the UK (EPSRC 2014). Other new designs include spinning disc (EPSRC 2014) and microwave technologies for solvent regeneration. The spinning disc technology involves an RPB and reboiler rotated on the same shaft. This design is currently developed in a UK EPSRC funded project (EPSRC 2014). It’s a first-of-its-kind design and presents a lot of structural and process design challenges. Microwave technology on the other hand, involves using microwave heating for solvent regeneration instead of steam. Industrial microwave heating is already a commercial technology (AMT 2016). However, for solvent regeneration, no previous study has been reported. New challenges expected include thermo-physical characterisation of the system among others.

### New solvents

New solvents with higher CO_2_ loading capacity and lower regeneration energy could significantly reduce the CAPEX and OPEX of PCC process. Emerging solvents with this potential could be classified as follows:

- Precipitating solvents: An example include amino acid salts such as potassium taurate among others. Techno-economic analysis of the DECAB process (Versteeg et al. 2003), a patented PCC process with aqueous amino acid salt solvent, indicated that the capital and operating cost is about half that of a similar capacity MEA process (Brouwer et al. 2005).
- Biphasic solvents: These solvents under regulated conditions undergoes liquid–liquid phase separation to give a CO_2_ lean and rich phases respectively (Raynal et al. 2011). Analysis of DMX™ process which uses biphasic solvents shows about 50% less regeneration energy and lower reboiler temperature compared to the MEA process.

These solvents are largely at an early stage development with TRL of 4 and not yet substantially proven for commercial deployment in PCC applications. There are also issues with them such as dealing with precipitates in the absorber for amino acid salts (Lerche 2012) and regulating phase change behaviour for biphasic solvents (Zhang et al. 2013) which are yet to be properly understood.

### Combination of new solvents and process

Emerging solvents are generally viscous and are difficult to handle efficiently in conventional packed bed designs. New packed designs must therefore be developed for these solvents. In a new project (started in Oct. 2016), ROLINCAP (CORDIS 2016), a consortium of 12 partners funded by EU seeks to develop specialized RPBs for biphasic solvents. Biphasic solvents have shown low regeneration energy requirement of about 2.4 GJ/ton of CO_2_ compared to about 4 GJ/ton of CO_2_ for MEA (Raynal et al. 2011). RPBs on the other hand have also shown good potential in replacing conventional packed beds.

## Conclusions

PCC based on chemical absorption is a near term technical option for commercial CCS deployment. The technology has been widely validated through pilot plant tests and different aspects of the technology have been investigated through modelling and simulation. Commercial products for modelling and simulation of such processes are now available. The technology can now be purchased off-the-shelf from different vendors namely Shell, Siemens, FLOUR and Alstom.

The technology is already widely deployed in industry but only one large scale commercial carbon capture plant is operating in the power sector. Regardless, the technology CAPEX and OPEX remains unacceptably high. Research in the past decade in this area have been targeted at making the technology more economically-attractive so as to drive its development and deployment. Predicted pathways for research include developing new processes based on PI technology, developing new solvents including precipitating and biphasic solvents and a combination of new processes and new solvents such as the ROLINCAP approach.
